# Level-weighted averaging in elevation to synchronous amplitude-modulated sounds

**DOI:** 10.1121/1.5011182

**Published:** 2017-11

**Authors:** Guus C. Van Bentum, A. John Van Opstal, Chaline M. M. Van Aartrijk, Marc M. Van Wanrooij

**Affiliations:** Department of Biophysics, Donders Institute for Brain Cognition and Behavior, Radboud University, Nijmegen, The Netherlands

## Abstract

To program a goal-directed response in the presence of multiple sounds, the audiomotor system should separate the sound sources. The authors examined whether the brain can segregate synchronous broadband sounds in the midsagittal plane, using amplitude modulations as an acoustic discrimination cue. To succeed in this task, the brain has to use pinna-induced spectral-shape cues and temporal envelope information. The authors tested spatial segregation performance in the midsagittal plane in two paradigms in which human listeners were required to localize, or distinguish, a target amplitude-modulated broadband sound when a non-modulated broadband distractor was played simultaneously at another location. The level difference between the amplitude-modulated and distractor stimuli was systematically varied, as well as the modulation frequency of the target sound. The authors found that participants were unable to segregate, or localize, the synchronous sounds. Instead, they invariably responded toward a level-weighted average of both sound locations, irrespective of the modulation frequency. An increased variance in the response distributions for double sounds of equal level was also observed, which cannot be accounted for by a segregation model, or by a probabilistic averaging model.

## I Introduction

Segregating sounds, and grouping them into perceptually distinct auditory objects, requires the brain to process distinct acoustic properties of a sound in parallel. The problem of sound-source segregation is known as the cocktail party problem, or auditory scene analysis (Alain and Arnott, 2000; Asari *et al*., 2006; Bregman, 1990; Cherry, 1953; McDermott, 2009; Roman *et al*., 2003; Wang and Brown, 2006). Physically, an auditory object comprises the spectraltemporal features that originate from the same sound source. For example, in the case of vibrating sources, like vocal chords, harmonic complexes have joint and synchronous comodulations in both time and frequency. In natural environments, it is extremely unlikely that multiple sources contain the exact same frequencies with identical onsets, offsets, and co-modulations, and this statistical fact can in principle be used as a prior to group sound features into distinct auditory objects (Bell and Sejnowski, 1995; Bregman, 1990; Darwin, 2008; Lee *et al*., 1998; Wang and Brown, 2006).

In addition to spectral and temporal disparities, the brain could potentially also use location information to segregate sound sources. In contrast to the visual system, which preserves location information of targets in spatially organized retinotopic maps, the auditory system has to rely on implicit acoustic cues for sound localization. Acoustic cues include interaural level and/or timing differences (ILD/ITD) for horizontal-plane localization, and pinna, head, and torsoinduced spectral-shape cues (referred to as directional transfer functions, or DTFs) for vertical-plane localization, and for resolving the cone of confusion (Blauert, 1997; Hofman *et al*., 1998; Middlebrooks and Green, 1991; Wightman and Kistler, 1989). The fact that a single sound source is confined to a unique location in space-time, and that in natural environments different sources do not originate from the same location, could theoretically further help the brain to segregate sounds.

Yet spatial hearing seems to play a minor role in sound segregation (Best *et al*., 2004; Bregman, 1990; Bremen and Middlebrooks, 2013; Schwartz *et al*., 2012); in the absence of non-spatial cues (such as harmonicity, or onset-disparity cues), it seems impossible to segregate sounds as different auditory objects in space. Instead, both in the horizontal plane (the stereophonic effect: Bauer, 1961; Blauert, 1997; but see Yost and Brown, 2013) and in the midsagittal plane (Bremen *et al*., 2010), the perceived location of synchronous sounds is directed toward a level-weighted average (WA) of the source locations. For the latter, weighted averaging occurs even when the spectral-temporal modulations of the sound sources are unrelated.

In contrast, Johnson *et al.* (2015) recently reported that synchronous sources in elevation can still be segregated if temporal envelope cues are present to suppress averaging. About half of their listeners successfully detected the up or down direction of an amplitude-modulated (AM, the target) broadband sound with low modulation frequencies (5–120 Hz), when another speaker from the opposite direction delivered a flat Gaussian white noise (GWN, the distractor). However, as the authors did not have their listeners determine the actual target sound location, it cannot be deduced whether or not the compulsory averaging behavior reported by Bremen *et al.* (2010) was indeed violated. For example, when a listener indicates a preference for an upward target direction, it is unclear whether she really perceived the sound at the veridical upward location, or whether there is merely a slight upward bias, which would still show as a weighted averaged response in an absolute localization task. Such a bias could potentially emerge while the amplitude modulation builds up, as a result of a time varying amplitude difference between the flat GWN and the AM noise.

To test whether particular amplitude modulations can indeed be used to accurately localize synchronous double sounds in elevation, we extended our previous work on weighted averaging (Bremen *et al*., 2010), by including modulation frequencies used by Johnson *et al.* (2015). Double-sound localization behavior in the free field was systematically studied under an open-loop localization paradigm. The target stimulus consisted of broadband GWNs with a sinusoidal amplitude modulation at either 5, 120, or 2000 Hz, while the distractor stimulus was not modulated. To test how sound level is weighted in the localization response, level differences between the stimuli were systematically varied between −10 dB (distractor louder) and +10 dB (target louder).

To study how segregation of synchronous double sounds in elevation through temporal envelope cues affects segregation, listeners also participated in an up-down discrimination paradigm (similar to Johnson *et al*., 2015). If participants can use temporal envelope cues to segregate sounds in the discrimination paradigm, a level-WA as in Bremen *et al.* (2010) will likely not be obtained in the localization experiments. Instead, participants would be able to localize the AM target sounds, even when the distractor sound would be louder. If, on the other hand, temporal modulation cues cannot be used to segregate sounds, one expects an averaged localization response, and chance discrimination performance for stimuli of equal levels.

## II Methods

### A Listeners

Eight participants (ages 20–39; mean 25; two females), all with normal hearing, as indicated by their audiometric curves (hearing thresholds <20 dB in both ears from 125 to 8000 Hz) took part in the experiments. Participants gave their full understanding and written consent prior to taking part in the experiments. Three participants contributed to this paper and were aware of the purpose of the study while the other participants were naive. All participants performed well in a standard single-sound GWN localization experiment prior to participating in the double-sound experiment (see Sec. III). Participants did not receive feedback about their performance, during or after the experimental sessions.

### B Setup

Experiments took place in a 3 × 3 × 3m sound-attenuated room, which had walls, floor, and ceiling covered with acoustic foam that absorbed sound-wave reflections above 500 Hz. All experiments were performed in complete darkness. Background noise level (measured with SLM 1352P, ISO-TECH level meter, RS Components BV, Haarlem) was 30 dBA. Sounds were presented from small, omnidirectional broad-range speakers (SC5.9, Visaton; Art. No. 8006, VISATON GmbH & Co. KG, Haan, Germany) which were mounted on an acoustically transparent spherical wire structure with radius 1.5 m. The participant was comfortably seated on an adjustable chair with the head positioned in the sphere’s center. Speakers were mounted within an orthogonal double-pole azimuth-elevation grid (Knudsen and Konishi, 1979) at approximately 15° intervals. On the cardinal axes, however, the speaker separation was 5°. No speakers were placed at elevations below −45°. Positive/negative azimuth angles indicate locations right/left from the listener’s midsagittal plane; positive/negative elevation angles refer to locations above/below the interaural axis of the participant.

Speaker locations were selected with a custom program, written in matlab (Mathworks, Natick, MA, version 2015b). The same program was used to record the head position and to play back sounds. Target speakers were controlled via two realtime processing units (RP2.1, Tucker-Davis Technologies, system 3, or TDT-3, Tucker Davies Technologies, Alachua, FL) and eight relay units (PM2R, from TDT-3). Sound levels were controlled by two active amplifiers (SA1, from TDT-3), and four programmable attenuators (PA5, from TDT-3).

Sounds were created offline in matlab and stored in a buffer on the RP2.1 before playback in each trial (at a sampling rate of 48828.125 Hz). Prior to the experiments, sound levels were measured for each stimulus type and speaker location to ensure equal-level presentation at the location of the listener’s head.

Head orientation in the localization tasks was recorded with the magnetic search-coil technique (Robinson, 1963; Van Wanrooij and Van Opstal, 2004), using a Remmel System 7 (Remmel Labs, Lacey, WA) for magnetic field generation and signal demodulation. A search coil was attached to a lightweight plastic glasses frame (glasses removed). From the nose bridge of this frame a small red laser dot was projected onto a small Styrofoam black plate (area about 1 cm^2^), positioned in front of the subject’s eyes, at about 40 cm distance at the end of a thin aluminum rod that also protruded from the frame. The laser dot helped the participant to fixate gaze, while freely turning the head in space. This method ensured the measurement of pure head-saccades, without the co-occurring saccadic eye-movements of natural gaze shifts. Three orthogonal pairs of square coils (6 mm^2^ copper wires, 3 m × 3 m) were attached to the room’s edges to generate the horizontal (*X*, 80 kHz), vertical (*Y*, 60 kHz), and frontal (*Z*, 48 kHz) oscillating magnetic fields, respectively, required for the search-coil method. The induced voltages in the search coil of the [*X*,*Y*,*Z*] movement signals of the head were demodulated, low pass-filtered (120 Hz cutoff), and sampled at 6 kHz (TDT-3 module RA16), before being stored on a disk. In each trial, three channels of 1500 ms duration of raw head-orientation data were recorded.

Psychometric responses (up/down) in the discrimination paradigms (described below) were recorded with a button box (RBOX, from TDT-3, connected to an additional RP2.1 module). Head position was also measured during discrimination experiments to exclude trials in which participants moved their head.

### C Sound stimuli

Sounds consisted of unmodulated GWN (bandpass filtered between 0.5 and 20 kHz), and 100% AM GWN. AM sounds were modulated by a sinusoidal envelope with modulation frequencies at 5, 120, or 2000 Hz. Sounds were generated offline and stored on a disk prior to playback. Whitenoise templates for AM and GWN sounds were generated in separate runs to avoid correlation between sounds. All sounds were given a ms sine-squared onset and cosinesquared offset ramp to prevent high-frequency transition artifacts (“clicks”).

### D Paradigms

#### 1 Head orientation calibration paradigm

To calibrate the search-coil signals into azimuth/elevation angles, a calibration experiment was performed first, in which the participant was asked to point the head (i.e., the laser dot) toward each of 24 evenly spaced LEDs that were mounted at the center of the speakers on the sphere structure. At each calibration trial, 200 ms of head-fixation position data were recorded. These data were used to train two feedforward neural networks that received the demodulated coil voltages as input, and yielded the corresponding azimuth/elevation angles as output (Neural networks toolbox, matlab). These trained networks were used to calibrate the voltage traces from the actual localization experiments (Van Wanrooij and Van Opstal, 2004).

#### 2 Double-sound localization paradigm

Participants were asked to fixate their gaze toward a green light emitting diode (LED) at (0,0)° (center of vision), and press a handheld button to initiate a trial. After the button press, there was a pause of 300–800 ms (drawn randomly from a uniform distribution), upon which the LED was turned off, followed 200 ms later by the presentation of the sound(s). This procedure was chosen to minimize the predictability of playback timing and to exclude potential after-effects of gaze-fixation. During playback either one or two sounds were played. Sound durations were 150 ms. In the double-sound condition, two sounds were played synchronously from two different locations on the midsagittal plane (at 0° azimuth). Sound levels were calibrated at 45, 50, 55, 60, 65 dBA, and were chosen such that between AM and GWN there was a level difference (Δ*L*) of [−10, −5, 0, +5, +10] dBA (positive level difference indicates that the AM sound was louder). Sound locations were chosen between −45° and +75° elevation, with separation angles between the speakers ranging between 15° and 75°.

Single sounds (both AM, GWN, and combined GWN+AM waveforms) were also included in the experiment to monitor single-speaker localization performance. In total, an experiment consisted of 720 double sounds (5Δ*L* × 3 modulation frequencies × 24 double-sound location configurations × 2 target/distractor configurations) and 119 single-sound trials, amounting to a total of 839 trials, divided over 4 recording sessions. Each session took around 20 min to complete. Single sounds were randomly interleaved with double sounds. Participants were instructed to localize the AM sound (the target) by making a fast and accurate goal-directed head saccade, hold the end position for about 1 s at the perceived location, and return to the fixation light straight ahead when it reappeared. Listeners were instructed to localize the GWN sound source, if they only heard that sound.

#### 3 Discrimination paradigm

In two experiments, participants had to press one of two buttons indicating the perceived direction (up/down at 20° above or below the horizontal plane) of an AM sound in a two-alternative forced choice paradigm. In the first experiment, AM sounds were presented with 5, 120, or 2000 Hz modulation frequencies and a modulation phase of 0 rad as targets, and the flat GWN acting as distractor. Sounds were 400 ms in duration. This experiment consisted of (2 locations × 3 modulation frequencies × 20 repeats =) 120 doublesound trials and (2 locations × 30 repeats =) 60 single sounds. In the second experiment, the target AM sounds had modulation phases of 0, *π*/4, *π*/2, 3*π*/4, *π*, 5*π*/4, 3*π*/2, and 7*π*/4 radians, and a modulation frequency of 5 Hz. This experiment consisted of 480 trials: (8 modulation phases × 2 locations × 10 repeats=) 160 single sounds, and (8 modulation phases × 2 locations × 2 sound types × 10 repeats =) 320 double sounds. For both experiments, participants were instructed to fixate their gaze at straight-ahead (at (0,0)° azimuth/elevation), and keep their gaze still during the trial. Either one or two sounds were presented at locations +20 and/or −20° elevation (azimuth zero). Target and distractor locations were pseudo-randomly varied between trials. Single-sound trials were pseudo-randomly interleaved with the double-sound trials. All sounds were presented at 55 dBA.

### E Data analysis

#### 1 Data selection

A custom-written matlab program was used to detect head saccades in the calibrated head orientation traces (e.g., Bremen *et al.*, 2010). The threshold for automatic head-saccade onset- and offset detection was set at 10°/s. We manually checked saccade profiles for irregularities (null-responses, anomalous profiles). Saccades that did not show clear, single peaked velocity profiles or saccades with reaction times well before sound offset (shorter than 150 ms) were discarded from further analysis.

#### 2 Bayesian analysis

To determine the influence of amplitude modulations on localization or discrimination performance, we wished to infer the contribution of the target location to the response in the localization task and the rate of choosing the target in the discrimination task. We chose to apply a Bayesian analysis (Gelman *et al*., 2013; Kruschke, 2014, 2010; Lee and Wagenmakers, 2014; Van de Schoot *et al*., 2014), as this provides a full posterior distribution on the joint probabilities of (combinations of) parameters (e.g., Kuss *et al*., 2005), rather than point estimates with parameter distributions obtained from *ad hoc* methods, such as bootstrapping (e.g., Bremen *et al.*, 2010).

##### a Localization model

**Fig. 1:**
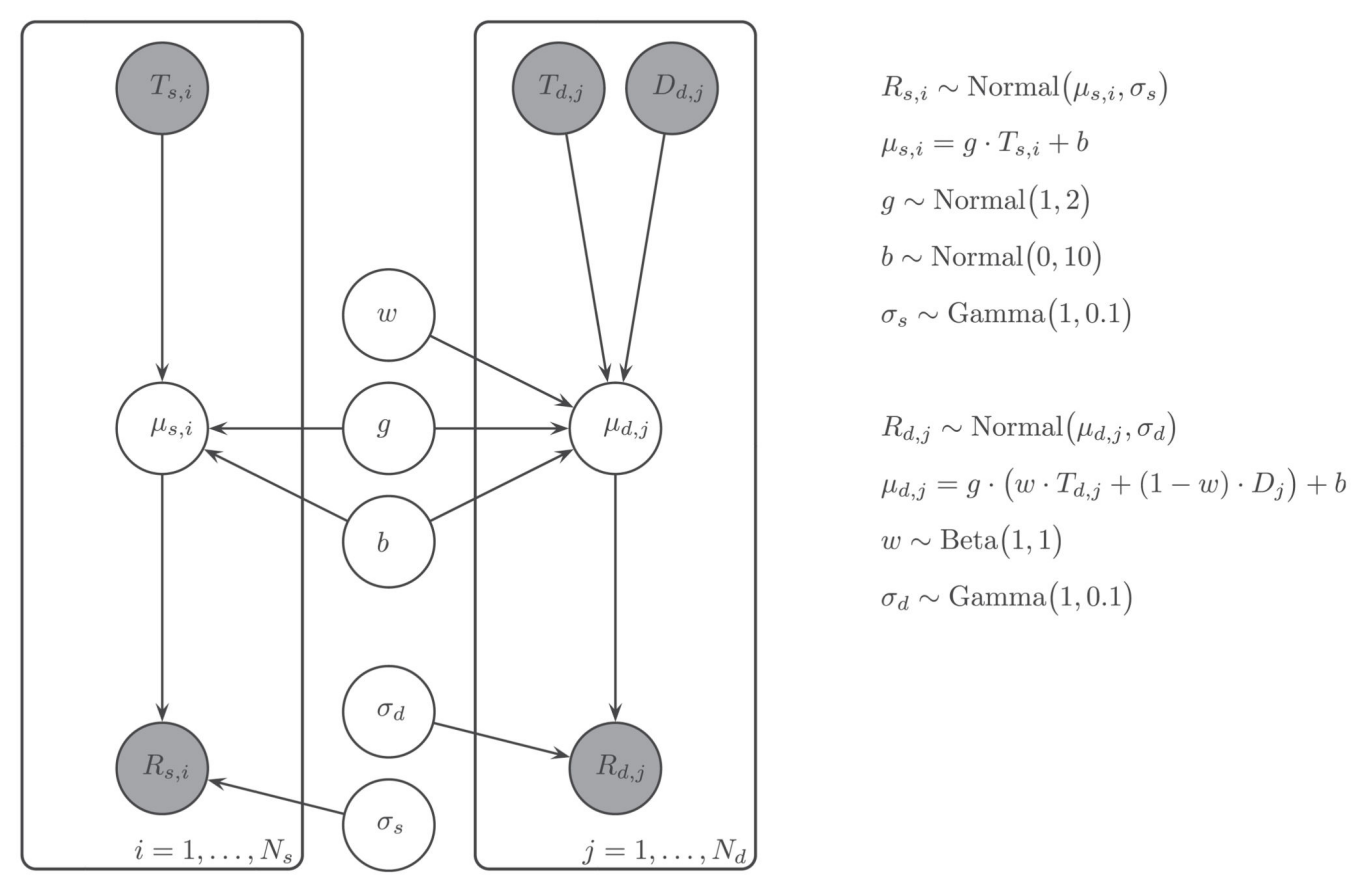
Graphical model representation for WA localization. The observed variables, target *T*, distractor *D*, and response *R* location are indicated by gray-shaded circles, while the latent parameters, weight *w*, gain *g*, bias *b*, and variability *σ* are indicated by non-shaded circles. Indices *i* and *j* indicate single- and double-sound trials, respectively, and are represented by encompassing plates. A mathematical description of the model equations and parameter distributions is shown on the right.

Figure 1 presents the graphical model we used to implement our model describing the localization behavior to double and single sounds. For double-sound localization data, we assumed the response for the *j*th double sound trial, *R*_*d*,*j*_, was normally distributed around a linear weighting function of the target location, *T*_*d*,*j*_, and distractor location, *D*_*d*,*j*_,

(1) $$\begin{array}{l} p\left(R_{d,j}|\mu_{d,j},\sigma_{d}\right)=\text{Normal}\left(\mu_{d,j},\sigma_{d}\right)\;\text{and}\;\mu_{d,j}\\ \;=g\left(w\cdot T_{d,j}+\left(1-w\right)\cdot D_{d,j}\right)+b \end{array}$$

where *μ*_*d*,*j*_ is the predicted value for the response to a double sound *R*_*d*,*j*_ (in degrees), with the subscript *j* denoting trial number, and where *σ*_*d*_ is the standard deviation of the responses around the prediction for double sounds (in degrees); *g* and *b* are the localization gain and bias (see below) and *w* is the weight of the target location. The weight, *w*, describes how much the target location contributes to the response relative to the contribution of the distractor. If *w* = 0, the response is independent of the target, if *w* = 1 the response fully depends on the target location with no contribution of the distractor, and if *w* = 0.5, the response is oriented toward the average of target and distractor locations.

For the single-sound localization data, it is assumed that the head-movement endpoints, denoted by response *R_s,i_*, were normally distributed around a linear function of the single target location, *T_s,i_*,

(2) $$p\left(R_{s,i}|\mu_{s,i},\sigma_{s}\right)=\text{Normal}\left(\mu_{s,i},\sigma_{s}\right)\;\text{and}\;\mu_{s,i}=g\cdot T_{s,i}+b$$

where *μ_s,i_* is the predicted value for the response to a single sound of *R_s,i_* (in degrees), with the subscript *i* denoting trial number, and where *σ_s_* is single sound response variability (in degrees).

Sound localization can typically be accurately described by a linear function (e.g., Corneil *et al*., 2002; Hofman and Van Opstal, 1998; Van Wanrooij *et al.*, 2009). Therefore, we modelled the predicted response value for both double and single sounds as a linear function with a slope, *g* (the *gain* or the sensitivity of a participant for changes in target location, dimensionless) and an intercept, *b* (the *bias* a participant had in localization, in degrees). Ideally, a participant has no localization offset, resulting in a bias *b* near 0°. For the gain *g* the ideal value is 1, indicating a one-to-one relationship between target and response location. We assume that the gain *g* and bias *b* parameters describe an individual’s localization behavior but that these remain identical for single sound and double sound trials (which were interleaved).

We placed proper approximations to non-informative distributions on all the parameters, so that they are all essentially flat over the values of interest. Specifically, we chose priors over localization bias *b*, localization gain *g*, and response variability *σ* that corresponded to the normal-hearing population (Corneil *et al*., 2002; Hofman and Van Opstal, 1998; Van Wanrooij *et al*., 2009). For the bias, this condition was met for a normal distribution with a mean of 0°, and a standard deviation of 10°. Similarly, for the gain the mean and standard deviation of the prior would then correspond to 1 and 10, respectively. For response variability *σ*, a Gamma prior was imposed for both single- and double-sound conditions, to ensure positive-only, real values. A uniform Beta prior was imposed on the weight, to ensure that *w* can take on any value between 0 and 1, but not outside that range.

Visual inspection of stimulus-response plots did not reveal bistable response behavior, where participants would localize either target or distractor with relatively high accuracy, but not in between both locations (as reported by Bremen *et al*., 2010; Yost and Brown, 2013). We therefore did not incorporate a bistable response mode in this model.

##### b Discrimination model

For the discrimination data, we assumed that the number of responses, denoted by *K*, in which a participant correctly identified the up- or down-direction of the AM target, was binomially distributed,

(3) p(K)=Binomial(θ,N),

where *θ* is the correct identification rate and *N* is the total number of trials. A *θ* value of 1 means that the participant always correctly indicated the direction of the AM sound, and a *θ* value of 0 means that the participant always incorrectly indicated the distractor direction. Since the rate parameter *θ* has to lie in between 0 and 1, a flat, uniform Beta prior was imposed on *θ*.

##### c MCMC analysis

Parameter estimation for the localization model and the discrimination model was performed using Markov chain Monte Carlo (MCMC) techniques with the JAGS program (Plummer, 2003; matlab implementation via matJAGS; Steyvers, 2011). Three MCMC chains of 10 000 samples were generated, of which the first 5000 were discarded as burn-in. Convergence of the chains was determined visually and by checking that the Gelman-Rubin-Brooks convergence diagnostic reached a value less than 1.1 (Brooks and Gelman, 1998; Gelman *et al*., 2013). Posterior distributions of parameters were sampled for all subjects and stimulus conditions (level difference, modulation frequency, modulation phase) separately.

##### d Statistical decision criteria

The Bayesian analysis yields a posterior distribution of all parameters of the underlying models. To summarize results, mean and 95% highest-density intervals (HDIs) of the posterior parameter distributions pooled across subjects were determined. For null hypothesis testing, Bayes factors (Jeffreys, 1961) were determined

(4) $${\text{BF}}_{10}=\frac{p\left(y|H_{1}\right)}{p\left(y|H_{0}\right)},$$

via the Savage-Dickey method (Dickey, 1971; *Wetzels et al*., 2010). The Bayes factor (BF) BF_10_ indicates how more likely the observed data *y* is under the alternative hypothesis *H*_1_ than under the null hypothesis *H*_0_. In the discrimination experiment, the null hypothesis is defined as *H*_0_ : *θ* = 0.5, whereas the alternative hypothesis is defined as *H*_1_ : *θ* ≠ 0.5. BFs of BF_10_ > 3 were taken to reflect a credible (cf. significant) difference between the alternative and null hypothesis. In general, Bayes factors can be interpreted and classified as substantial (3 < BF_10_ < 10), strong (10 < BF_10_ < 30), very strong (30 < BF_10_ < 100), and decisive (BF_10_ > 100) evidence (Jeffreys, 1961).

## III Results

### A Sound localization

**Fig. 2:**
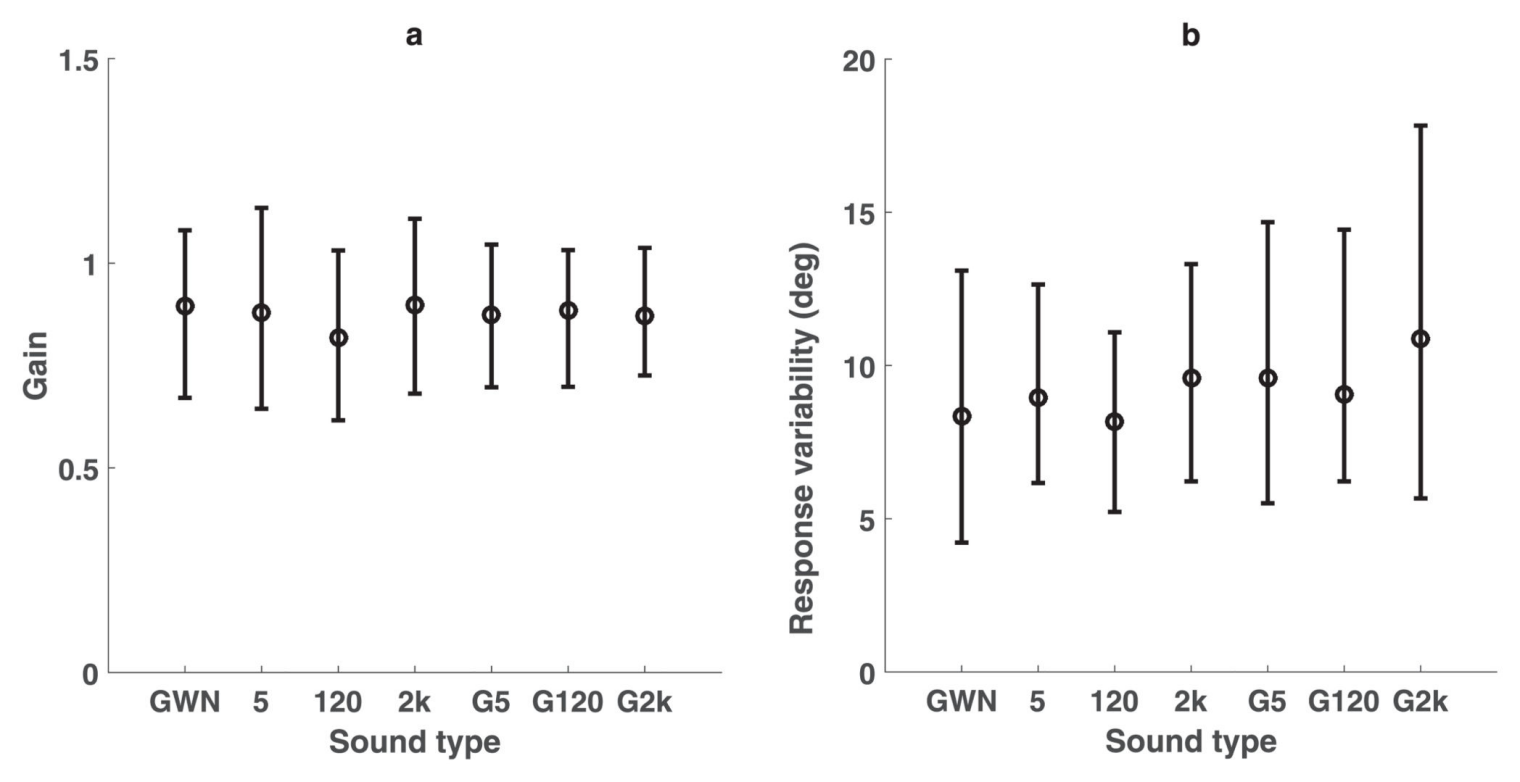
Single-sound localization performance for seven different sound types, shown as group-level statistics for seven listeners. GWN indicates static white noise, “5” indicates white noise with 5 Hz amplitude modulation, and “G5” indicates superimposed GWN and 5 Hz AM sound. Same conventions for the 120 Hz and 2 kHz AM noises. (a) Group level gains for different sound types. (b) Group level response variability, *σ*_*S*_ (deg), for the different sound types. In both panels, error bars indicate 95% HDI. Participants responded similarly to all different sound types.

Localization performance for responses toward the flat GWN and the AM-noises in both single- and double-sound trials were assessed by applying the Bayesian model described in Sec. II E 2. For single sound trials, either AM-noise or GWN was presented in isolation, or superimposed on the same speaker at all locations used in the double-sound trials (see Sec. II D 2). Participants could localize single-sound sources well (Fig. 2); gain [Fig. 2(a), as calculated using Eq. (2)] mean values ranged from 0.82 to 0.90 and response variability *σ* [Fig. 2(b)] mean values ranged from 8.2 to 10.9°, indicating accurate and consistent localization behavior, respectively. Both localization measures were about the same for all seven different sound types.

In the double-sound condition, both AM and GWN sounds were presented synchronously at different locations. The listener was instructed to localize the AM sound, while ignoring the unmodulated GWN.

**Fig. 3:**
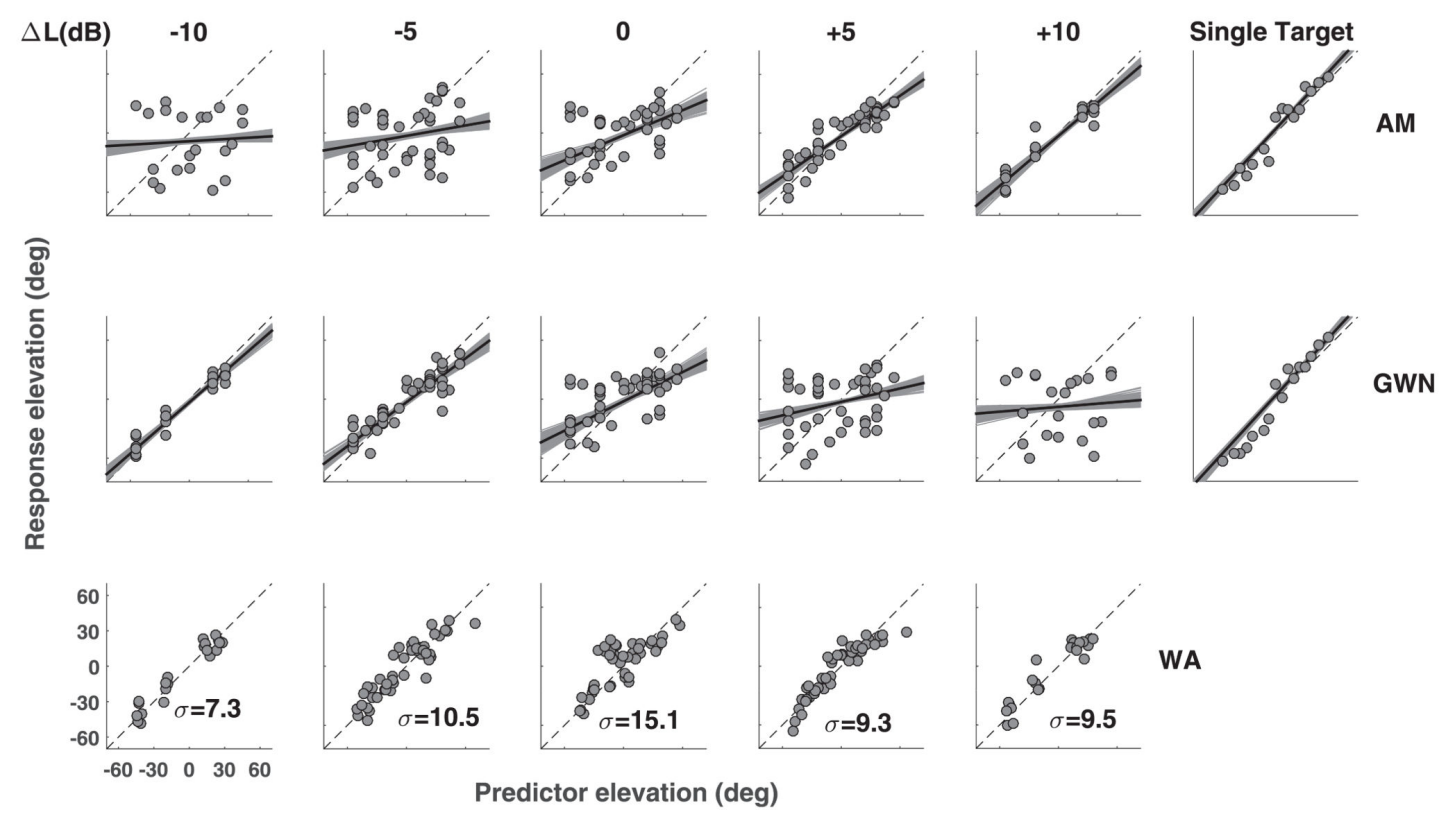
Double-sound predictor-response plots for participant 8. Gray dots correspond to individual responses, and the linear regression results to the black (mean) and gray (95% HDI) lines. All plots feature the 120 Hz AM sound as target. Rows indicate different predictor locations, columns indicate level difference (Δ*L*) between target and distractor sound. Top row: target (AM noise) versus response. Middle row: distractor (flat GWN) versus response. Bottom row: WA prediction [Eq. (1)] versus response. Response variabilities ***σ*** (deg) are shown in each subplot. Rightmost column features single target localization responses toward AM (top row) and GWN (middle) sounds.

To test how well participants performed this task, the gain, bias, and relative contributions (weight *w*) of the target location (AM) and the distractor location (GWN) to the response location [Eq. (1)] were calculated. The double-sound localization results indicated that at single participant level, stimulus-response relations were level dependent (Fig. 3; results for subject 8, shown for 120 Hz AM). For negative Δ*L* values, this subject showed a high gain for the distractor response (Fig. 3, top row, left-most panel), and, conversely, a low gain for the target-responses (Fig. 3, left-most panel, center row). For positive level differences, both relations featured opposite behaviors (Fig. 3, “+10” panels top and center row). Now the distractor-response relations had low gains, whereas the target-response regressions resulted in high gains. To test whether a level-WA of target and distractor location could serve as a better predictor for the localization response for all conditions, the WA prediction of Eq. (1) was calculated. We observed that for all Δ*L* values, single participant data showed little variation in gain, with values ranging between 0.88 and 1.05 (Fig. 3, bottom row). The lowest value was obtained for Δ*L* = 0 dB, which also induced the largest response variability.

**Fig. 4:**
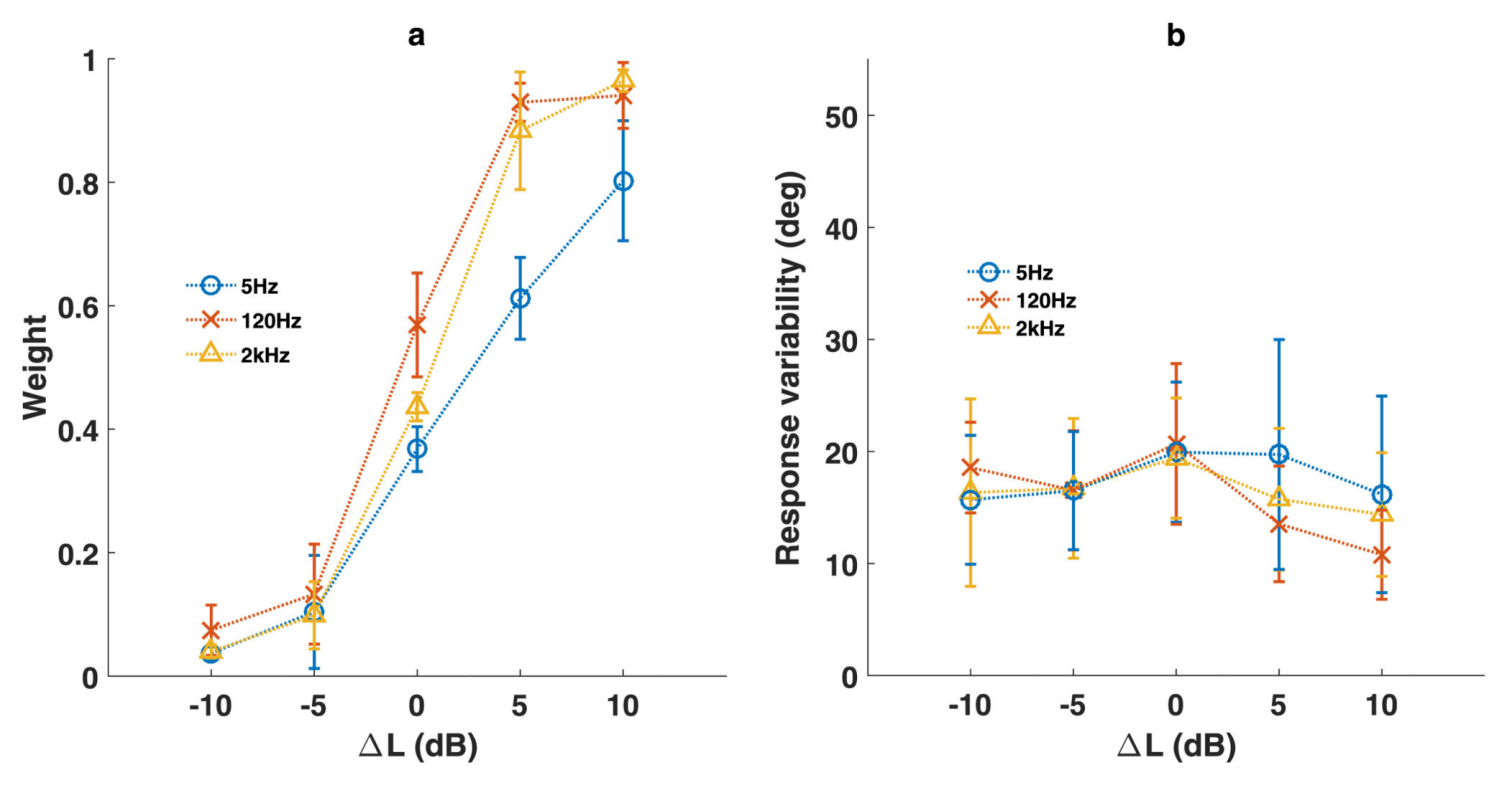
(Color online) Double-sound predictor weights (a) and response variabilities (b). (a) Predictor weights *w* in WA response location, as a function of level difference (Δ*L*). Different shades indicate the three modulation frequencies (5, 120, 2000 Hz). Values averaged over participants, error bars indicate standard deviation. (b) Response variability (*σ_d_*, in deg) around model prediction for double sounds, as a function of level difference (Δ*L*). Different modulation frequencies indicated in different colors. Values averaged over participants, error bars indicate standard deviation. Note largest variability for averaging responses obtained at Δ*L*=0 dB (120 and 2 kHz) and 5 dB (5 Hz).

Averages of both regression weights [Fig. 4(a)] and response variability [Fig. 4(b)] showed that for all modulation frequencies, target localization is systematically influenced by level difference. Target-location weights increased monotonically with increasing Δ*L*, for all three AM stimuli [Fig. 4(a)]. For −10 dB, the weight was nearly zero, indicating no influence of target sound on the response. For +10 dB, the weight was nearly one, indicating a large influence of the target on the response. At 0 dB, weights for 2 kHz and 120 Hz stimuli were close to 0.5, indicating averaging of target and distractor. The weights for the 5 Hz stimuli were lower (in the 0, +5, and +10 dB conditions) than for the 120 Hz and 2 kHz stimuli.

For the weighted-average model, response variability was consistent for different modulation frequencies, as well as for different Δ*L* conditions[Fig. 4(b)]. Response variability in double sound conditions was generally higher than for single sound conditions [cf. Fig. 2(b)]. As observed for weights, there is a difference between the response variability curves 5 Hz and 120 Hz/2 Khz stimuli. Peak variability for 120 Hz/2 kHz is observed at 0 dB, whereas peak values for 5 Hz are observed at +5 dB.

### B Discrimination of AM noises with different modulation frequencies

**Fig. 5:**
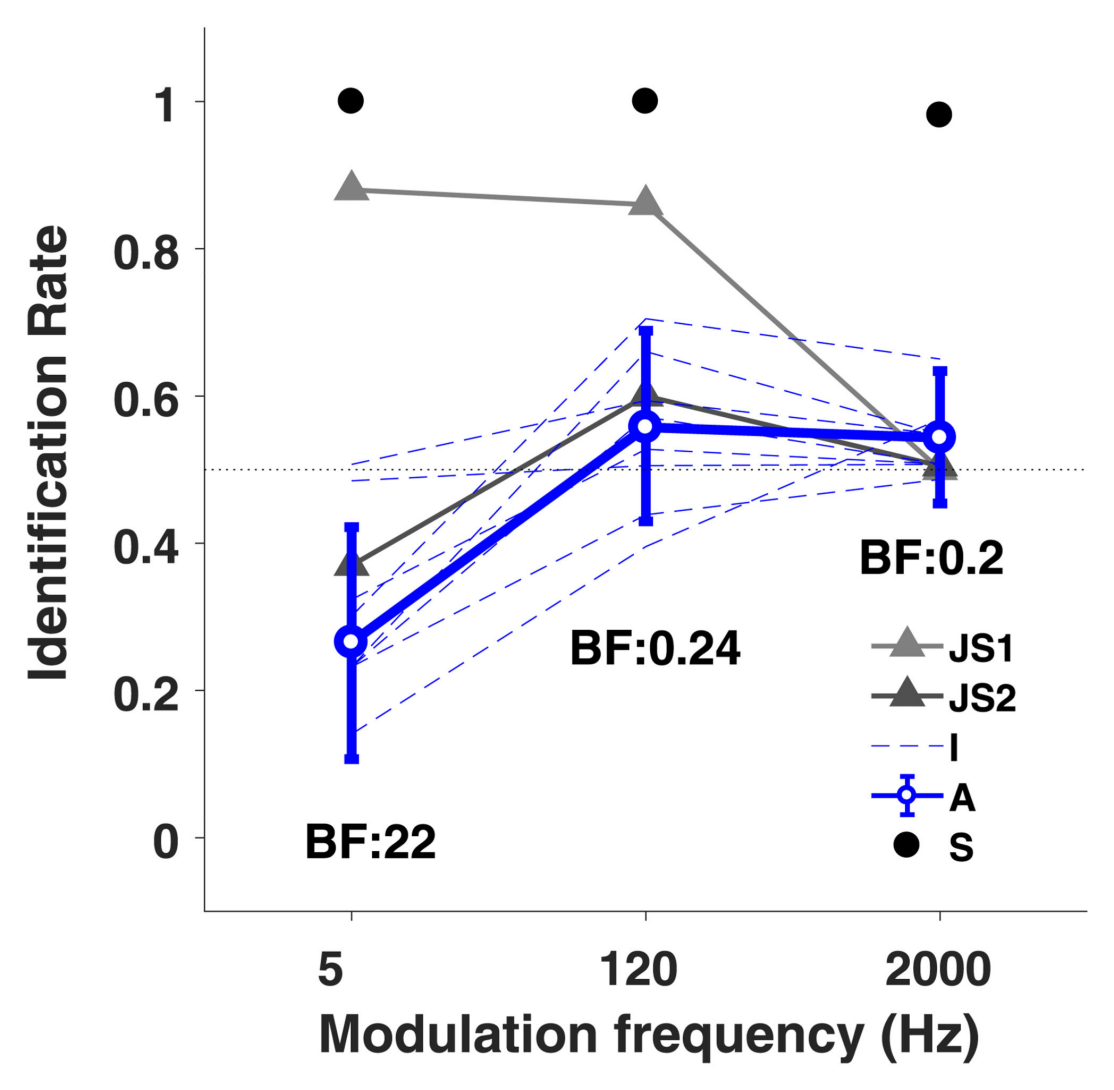
(Color online) Results of discrimination experiment, pooled for all subjects. AM identification rate (*θ*) is shown for three modulation frequencies. Thick blue line (“A”) shows group level identification rates from the current experiments. Individual subject data are shown in dotted blue (“I”). Group level single-target identification rates are shown as black dots (“S”). Error bars indicate 95% HDIs. Numbers below data points indicate Bayes factors per modulation frequency. The 5 Hz modulation frequency data show that, despite task instructions, participants indicate GWN sound location as the AM sound location. Data “JS1” and “JS2” (gray) are identification results for the two distinct responder groups in Johnson *et al.* (2015). Note that these results are not provided as identification rates *θ*, but by the relative identification score, K/N and that error bars are therefore missing.

In the modulation-frequency discrimination experiment, all eight listeners were able to identify the target speaker (up or down at +/−20° elevation) at ceiling performance regardless of modulation frequency (Fig. 5, black dots) if only a single target AM sound (with modulation phase zero) was presented. In contrast, in the presence of a concurrent distractor of equal level (55 dBA), participants identified the target AM speaker around chance level for any modulation frequency (Fig. 5, blue). For 5 Hz AM sounds, participants even responded to the distractor speaker with a higher-than chance probability (95% HDI does not contain *θ* = 0.5 value, BF = 24). At the higher modulation frequencies (120 and 2000 Hz) performance was at chance level (95% HDI contains *θ* = 0.5 value and Bayes factors are smaller than 1, indicating more evidence in favor of the null hypothesis *θ* = 0.5). None of the participants correctly identified the target at rates of up to 0.9, as described earlier by *Johnson et al.* (2015) for a subgroup of listeners for the lower (5–120 Hz) modulation frequencies (for comparative purposes, their data are shown in Fig. 5, gray lines).

### C Discrimination of AM noises with different modulation phases

**Fig. 6:**
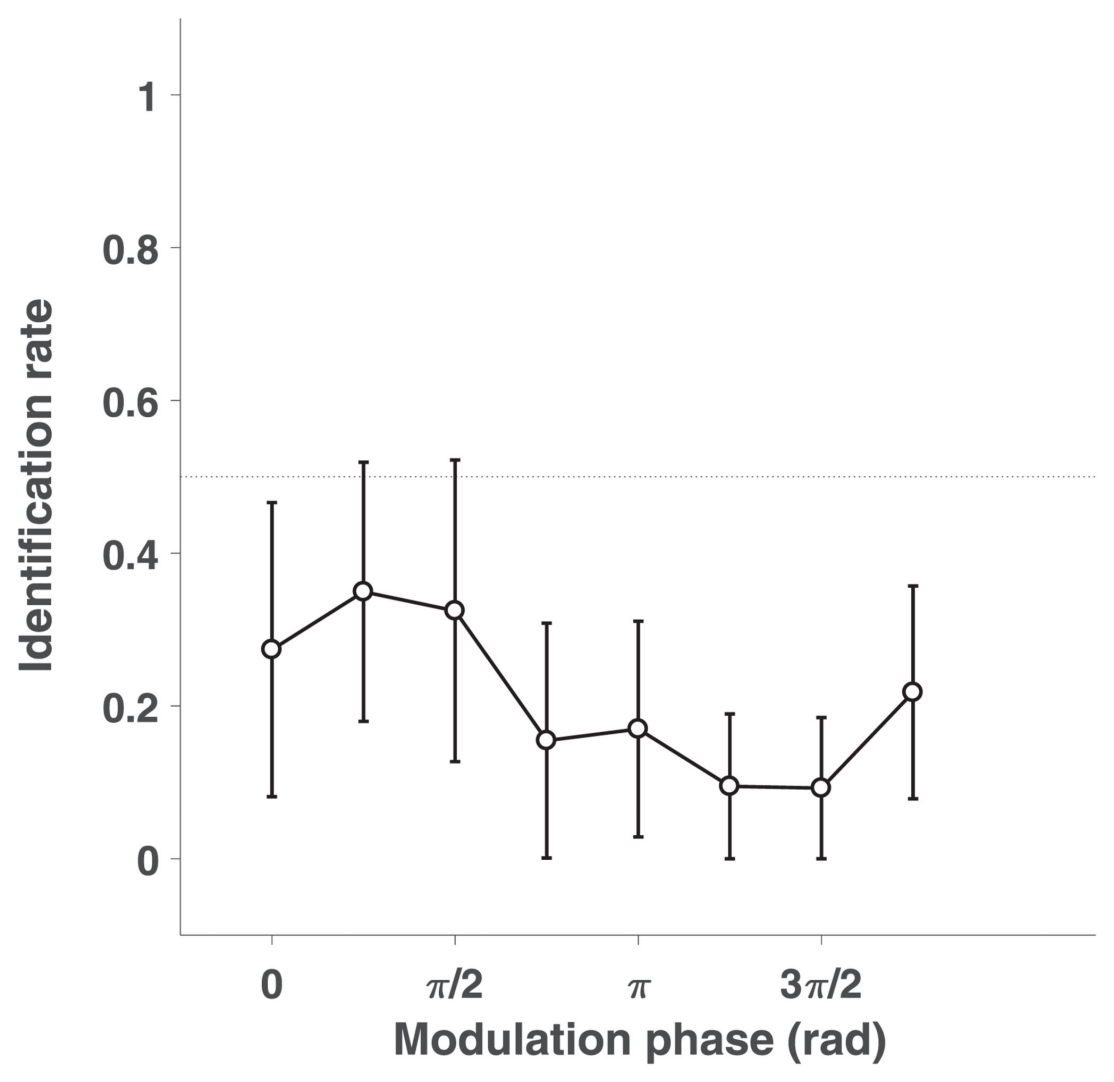
Identification rates toward phase modulated 5 Hz AM + static GWN stimuli. AM identification rate *θ* as a function of modulation phase shown in black. Error bars indicate 95% HDIs.

In the modulation phase discrimination experiment, none of the eight participants could identify a target 5 Hz AM sound location above chance, regardless of the modulation phase (Fig. 6, black curve). Instead, the average rate *θ* across participants was biased for every modulation phase toward the GWN distractor (mean identification rates *θ* < 0.5, 95% HDIs do not overlap with *θ* = 0.5 except for phase = 1/4*π*, BF > 8, not shown in the figure). This indicates that listeners consistently and wrongly identified the 5 Hz AM sound in the direction of the GWN distractor. Interestingly, the identification rate also varied in a systematic way with the modulation phase (see Sec. IV).

## IV Discussion

### A Summary

Broadband synchronous sounds presented in the midsagittal plane evoke a spatial percept that is determined by relative sound levels and spatial separation, rather than by task instructions. Our experiments demonstrate that additional amplitude modulations do not contribute to spatial segregation of synchronous sound sources. The results from our localization experiments confirm that orienting responses toward double-sound sources are best described by level-WAs of the target and distractor locations [Eq. (1), Figs. 3 and 4]. Localization behavior was insensitive to the modulation frequency, except for the lowest modulation frequency employed in this study (5 Hz), which resulted in a localization bias towards the distractor (static GWN) locations (Fig. 4).

The discrimination experiments showed that participants were unable to correctly indicate the direction of the target (AM) sound, when presented synchronously and at equal level with the distractor (flat GWN) at the different modulation frequencies. At the 5 Hz amplitude modulation, we obtained a strong bias towards the distractor GWN sound (Figs. 5 and 6), which systematically varied with the modulation phase (Fig. 6). As will be argued below, this phase-dependency may be due to ongoing power differences between the 5 Hz AM and the flat GWN stimulus.

At higher modulation frequencies (120 Hz and 2000 Hz) these ongoing level differences average out, and with it the response bias, as for these stimuli subjects invariably responded at chance levels. We therefore conclude that the compulsory WA model proposed by Bremen *et al.* [2010; Eq. (1)] also accounts for the discrimination data to broadband synchronous sounds.

### B Amplitude modulation as a cue for spatial segregation

Our experiments do not confirm the results from Johnson *et al.* (2015), who found that half of their participants could successfully indicate the direction of AM sounds in the same discrimination paradigm, especially for the lower modulation frequencies (≤120 Hz, 5 Hz data shown in Fig. 5). Since both studies obtained results from comparable sample sizes (*N* = 9 vs *N* = 8 in our study), and employed identical stimuli, it is unlikely that we would not have encountered participants with high positive identification scores.

An interesting similarity in the results of both studies is obtained for the group of participants who were biased toward the *distractor* sound for the 5 Hz modulation frequency. We here showed, by systematically varying the phase of the modulation envelope, that the psychometric parameter *θ* (the identification rate) was highly phase dependent (Fig. 6). Although the acoustic power averaged over the full 400 ms sound duration (two complete AM periods) was the same for all 5 Hz AM sounds in our experiment, the different modulation phases resulted in clear differences in the initial stimulus power during the first tens of milliseconds. Earlier studies have indicated that the human auditory system needs about 40–80 ms of broadband acoustic input to accurately localize source elevation (Hofman and Van Opstal, 1998; Vliegen and Van Opstal, 2004). Thus, differences in the initial acoustic power of the stimuli could have determined the perceived elevation of the double-sounds, rather than the overall acoustic power of the stimuli. If so, the identification rate would co-vary with the phase of the AM noise for the low-frequency stimuli, which was indeed observed when the time window was in the order of about 50–100 ms (not shown).

In contrast to the phase-dependent identification rate for the low modulation frequency, participants showed no bias toward the GWN sounds for the higher modulation frequencies. These stimuli had much steeper onset ramps that do not influence the processing of elevation cues, and the putative analysis window of the auditory system (extending to several tens of ms) would average out across multiple modulation periods, yielding no systematic phase-dependent level differences.

In this paper, we used both free-field localization and forced-choice tasks. For the participants’ performance in either task, we found no evidence for segregation. Apart from the behavioral task, conditions in both experiments were substantially different, which further supports the hypothesis that amplitude modulations do not aid sound segregation of synchronous stimuli. The number of speaker configurations (75 in the free field, only two in the forced choice task), the sound durations (150 ms in the free field, 400 ms for the forced-choice task), and the different sound levels used in the two paradigms, also give further weight to this hypothesis. According to Bregman (1990) differences in sound duration could possibly play a role in sound segregation, but whether the relatively long durations used in our study were sufficient for a potential segregation in elevation remains to be tested. We also verified whether the participants’ head position changed in the forced-choice experiments, which could potentially provide additional dynamic segregation cues during stimulus presentation, but we found no evidence for improved performance due to small head movements.

We hence conclude that amplitude modulations, as mentioned earlier in Bremen *et al.* (2010), cannot be used to drive spatial segregation in elevation when the auditory system is presented with synchronous sounds.

### C Weighted averaging

In our localization experiments, the weighted-average model [Eq. (1)] matches, or outperforms, all single target-based predictions [Eq. (2); Fig. 3]. Localization performance at the two highest modulation frequencies (AM at 120, 2 kHz) was very similar. Results for low-frequency (5 Hz) modulations showed similar trends for target-weights, yielding a monotonic weight progression with increasing Δ*L* (Fig. 4). We noted that for the 5 Hz modulations, the curves were shifted to the right, so that points of equal target/distractor model variability and equal weight values (*w* = *0.5*) occurred at Δ*L*=+3 dB, instead of at Δ*L* = 0 dB, as observed for the higher modulation frequencies. We attribute this shift to the initial level differences between the 5 Hz AM sound (presented at phase 0) and the GWN sound during the initial part of the stimulus, as also observed in our discrimination data (see above, and Fig. 6).

Although the weighted-average model best explains the data, at Δ*L* = 0 dB (or, equivalently, at Δ*L* =+3 dB for the 5 Hz AM stimulus) we noted an increase in the model’s response variability around the WA predictions [Fig. 4(b)]. However, this difference fell within the 95% HDI range of the other level difference conditions. This increased variability (and associated decrease in gain) was also observed by Bremen *et al.* (2010), and thus appears to be independent of the target/distractor sound combinations. Possibly, the perceived sound location becomes spatially more diffuse in these conditions (see also below). Contrary to an earlier suggestion (Bremen *et al*., 2010), we obtained no conclusive evidence for bi-stable response behavior, as the response distributions were single-peaked.

In the current experiments, all synchronous stimuli were presented within the midsagittal plane, where the ITD/ILD cues between target and distractor are negligible. There is no compelling reason to assume that the median plane would have a special status in the brain over any other potential source directions, as the distribution of sound sources in the natural world will hardly ever be located exactly in this plane. In other words, typical sound sources will be endowed with both a nonzero azimuth and elevation coordinate, and thus the classical stereophonic effects on the basis of ILD/ITD differences for the azimuth components of double sounds will nearly always be present. However, whether, and how, the stereophonic azimuth effects and the averaging effects for the median plane interact, is not known. Followup experiments with double stimuli distributed across the two-dimensional directional space will be required to investigate this phenomenon in more detail.

### D Discrimination versus localization

The discrimination paradigm forces listeners to choose for either the upward or downward stimulus location, regardless of their absolute spatial percept. This paradigm cannot disclose whether subjects perceived the sound at the veridical location, or whether the spatial percept would only be slightly biased into the veridical direction of the sound source. As a result, the discrimination results cannot readily discard the weighted-averaging hypothesis, even when subjects would consistently indicate the correct direction of the target (which they do not, see Figs. 5 and 6). A continuous measure (pointer) of localization performance would be required to estimate the absolute spatial percept as a function of the acoustic parameters (timing, level, spectrum, modulation frequency). Indeed, although the results of our discrimination paradigm (Fig. 6) can still be understood from the level-weighted averaging model, the compulsory nature of level-weighted averaging follows from our orienting experiments.

### E Object formation and spatial segregation in the median plane

Acoustic interactions between sources (superposition of sound waves) result in the loss of spatial cues (DTFs) to segregate sounds in the median plane. Our data showed that added temporal information (amplitude modulation) does not lead to segregation. Possibly, amplitude modulation could lead to object formation, but not to correct localization in the median plane. This could be explained by the fact that the neural origins of segregation and localization may be very different. It could also be that segregation in the median plane is canceled by some form of likelihood averaging, or by interactions of spatial maps in the brain.

The human ability to spatially segregate sound sources is then still an open question, if it is not modulated by amplitude modulation. The type of cues used to segregate sounds, and subsequently localize them, is not fully understood. Possibly temporal onset cues, or binaural cues, are the defining factor in this process. Double-target localization experiments that address onset, binaural, temporal, harmonicity, and other cues might give a more definitive insight.

### F Conclusion

We conclude that the internal prior of the brain that sounds from independent, spectrally overlapping sources never occur in perfect synchrony cannot be overcome by providing additional temporal information in one of the stimuli. As a result, the auditory system merges the two sounds into a single auditory object, the spatial extent of which results to be broader than for single-sound sources, and at a mean location that is a level-WA of the individual stimulus locations. These properties cannot be simply explained by mere acoustic wave interference in space, before the acoustic input reaches the ears. The weighted averaging process appears to be compulsory, and has some interesting resemblances to visuomotor processing (the “global effect”; Findlay, 1982; Ottes *et al*., 1984). Note also that the observed increased variability of averaging responses is not in line with Bayesian cue-combination, which would predict more precise averaging responses, with less variance (Alais and Burr, 2004). We instead speculate that weighted averaging reflects neural mechanisms that involve interactions within spatially organized maps, in combination with internal assumptions (learned priors) about natural acoustic environments.
